# An exact solution for the free-vibration analysis of functionally graded carbon-nanotube-reinforced composite beams with arbitrary boundary conditions

**DOI:** 10.1038/s41598-017-12596-w

**Published:** 2017-10-10

**Authors:** Zeyu Shi, Xiongliang Yao, Fuzhen Pang, Qingshan Wang

**Affiliations:** 1 College of Shipbuilding Engineering, Harbin Engineering University, Harbin, 150001 PR China; 2 State Key Laboratory of High Performance Complex Manufacturing, Central South University, Changsha, 410083 PR China; 3 College of Mechanical and Electrical Engineering, Central South University, Changsha, 410083 PR China

## Abstract

We present an exact method to model the free vibration of functionally graded carbon-nanotube-reinforced composite (FG-CNTRC) beams with arbitrary boundary conditions based on first-order shear deformation elasticity theory. Five types of carbon nanotube (CNT) distributions are considered. The distributions are either uniform or functionally graded and are assumed to be continuous through the thickness of the beams. The displacements and rotational components of the beams are expressed as a linear combination of the standard Fourier series and several supplementary functions. The formulation is derived using the modified Fourier series and solved using the strong-form solution and the weak-form solution (i.e., the Rayleigh–Ritz method). Both solutions are applicable to various combinations of boundary constraints, including classical boundary conditions and elastic-supported boundary conditions. The accuracy, efficiency and validity of the two solutions presented are demonstrated via comparison with published results. A parametric study is conducted on the influence of several key parameters, namely, the L/h ratio, CNT volume fraction, CNT distribution, boundary spring stiffness and shear correction factor, on the free vibration of FG-CNTRC beams.

## Introduction

Carbon nanotube (CNT)-reinforced composites have shown outstanding physical, mechanical, thermal and electrical properties over traditional structural materials, drawing interest from numerous researchers. CNTs are recognized as well suited to reinforce polymer composites due to their high elastic modulus and tensile strength and low density^1–4^. As a result, enthusiasm for research activities involving CNTs has been ignited in recent years. Wagner *et al*.^5^ performed tensile experiments on multi-walled carbon nanotubes (MWCNTs) and analysed the transformation of the elastic modulus and the break stress of nanoscale reinforced composites. Qian *et al*.^6^ developed homologous research two years later, noting that the addition of only 1% MWCNT to polystyrene significantly improved its polymeric mechanical properties. Fiedler *et al*.^7^ demonstrated the superiority of the CNTs as nanofillers in polymers and suggested that a distribution of CNTs should be concentrated or dispersed to realize the best possible properties. Han^8^ and Wan^9^ found that the introduction of low volume fractions of nanotubes in matrices can result in notable strengthening of the composite properties. Relative to micron-scale counterparts, the interfacial regions between the nanoparticles and the matrix are strongly reactive. Coleman *et al*.^10^ compared mechanical properties and various manufacturing processes of single-walled carbon nanotube (SWCNT)-reinforced composites to MWCNT-reinforced composites. However, the collective problems of dispersion and stress transfer still lack solutions.

To overcome these issues, functionalization through chemical procedures, for example, has been adopted by researchers. Functionally graded (FG) materials, which act as primitive thermal barrier materials in the aerospace industry, are widely known for their smooth and continuous variations in material properties. In this way, the integration of single materials with different properties can be improved, and the advantages of material properties can be combined^11–13^. Shen^14^ in 2009 first proposed a new distribution form with CNTs distributed in an FG manner in the matrix; the volume fraction of CNTs was assumed to vary along the thickness direction. The issue of nonlinear bending behaviour of functionally graded carbon-nanotube-reinforced composite (FG-CNTRC) plates was investigated in which a transverse uniform or sinusoidal load in thermal environments was taken into consideration. The results revealed that mechanical, electrical and thermal properties varied considerably with the FG distribution of CNTs.

CNT-reinforced composites, which are increasingly used, can be formed into structures such as beams, plates and shells. Beams are a fundamental and significant structure in comprehensive engineering applications in the fields of marine, aerospace, civil, and mechanical engineering, among others areas. In this regard, various studies have focused on dynamic characteristics analysis of beam structures to guide structurally reliable design of such engineering applications^15–20^. Incorporating first-order beam theory, Yas and Samadi^21^ adopted the generalized differential quadrature method to analyse the issue of vibrations and buckling of carbon-nanotube-reinforced composite (CNTRC) beams on elastic foundations. Four different CNT distributions were considered, and the material properties of the nanocomposites were obtained from the rule of mixtures. In light of the von Kármán geometric nonlinearity displacement–strain relationship and Euler beam theory, the linear and nonlinear vibration behaviours of CNT-reinforced FG composite beams were presented by Rafiee *et al*.^22^. Numerical results showed that an increase in the CNT volume fraction led to an increase in the nonlinear-to-linear frequency ratio and the natural frequencies. Lin and Xiang^23,24^ investigated the free-vibration characteristics of CNT-reinforced beams with soft-clamped and hard-clamped boundary conditions, with uniformly distributed (UD) CNTs and FG distribution being considered. The model was established based on first-order and third-order shear deformation elasticity theories and solved using the polynomial Ritz method. The research showed that ratios of nonlinear-to-linear frequency parameters and natural frequencies based on first-order and third-order shear deformation elasticity theories with soft-clamped boundary conditions showed manifest deviations. Ke *et al*.^25^ investigated the nonlinear vibration characteristics of FG-CNTRC beams with various boundary conditions using a direct iterative method. The influences of the vibration amplitude, volume fraction of CNTs, ratio of length to thickness, boundary conditions and CNT distribution were taken into account to characterize nonlinear vibration in the beams. The response of CNT-reinforced FG composite beams under low-velocity impact was first analysed by Jam and Kiani^26^. On the basis of first-order beam theory, the behaviour of FG-CNTRC beams exposed to the impact of a small mass was solved by means of the conventional polynomial Ritz method and the Runge–Kutta method. The peak contact force was found to be proportionate to the volume fraction of CNTs and inversely proportional to the temperature, while the contact time behaved oppositely.

With the rapidly increasing industrial use of composite materials, various numerical tools and theories have been promoted to analyse the mechanical behaviour of composite structures^27–30^. The problem of nonlinear vibration of composite plates reinforced by CNTs was presented by Wang^31^. The governing equation of the CNTRC plate was derived according to higher-order shear deformation theory, and the theoretical model was solved using the improved perturbation technique. Zhang *et al*.^32^ focused attention on the free-vibration characteristics of CNT-reinforced FG composite triangular plates and adopted the element-free IMLS-Ritz method based on first-order beam theory. In view of the first order shear deformation theory, Rafiee *et al*.^33^ employed the Galerkin method and the harmonic balance method to investigate initially imperfect piezoelectric composite plates reinforced by SWCNTs. The vibrational characteristics and buckling of an FGM microplate with two different supports were studied by Ke *et al*.^34^. Wang^35,36^ developed a unified semi-analytical approach and applied it to the issue of free-vibration analysis of FG-CNTRC structures of revolution, including spherical panels and doubly curved shells. The differential quadrature method and the Mindlin plate theory were applied in this research to enable scientific conclusions to be drawn. Based on the FEM and two types of shear deformation theory, Yas and Heshmati^37^ established an analytical model of a FG-CNTRC beam subjected to a moving load.

Numerous studies have been conducted to illustrate the vibrational characteristics of CNTRC beams. Nevertheless, the investigations mentioned above are limited to several representative boundary conditions. A diversity of boundary restraints leads to notable changes in the free-vibration characteristics. Relatively little study has addressed the free vibration of CNT-reinforced beams with elastic supports, although various possibilities of boundary conditions appear in engineering practice. Furthermore, most actual solution procedures are customized to restricted forms of certain classes at both ends of the beam. Consequently, existent contributions are urgently sought not only to guide engineering applications but also to enhance complementary research.

Aiming at satisfying practical needs, in this investigation, a unified and satisfactorily accurate method is presented for the free-vibration analysis of FG-CNTRC beams with arbitrary boundary conditions, including various classical boundary conditions and elastic supports. The modified Fourier method was first proposed by Li to analyse the vibration of a beam^38^ and was subsequently extended to plates^39–47^ and shells^48–53^. The two functions of displacement and two functions of rotation are expressed as a linear combination of an original Fourier cosine series expansion and two complementary auxiliary polynomial functions. The supplemental items are introduced to remove the potential discontinuities of displacement components and derivatives of displacement functions at the ends of the beam and to accelerate convergence of the solution procedures. Arbitrary boundary conditions can be conveniently achieved through assigning the appropriate stiffness to four sets of boundary springs at each edge of the CNTRC beam without updating the solution procedure.

In the present work, a strong-form solution procedure of the modified Fourier method is proposed and used to solve generalized eigenvalue problems directly by submitting a modified Fourier series to the governing equations and the boundary conditions. In addition, the results obtained from the Rayleigh–Ritz technique associated with the modified Fourier method are presented here as a weak-form solution for comparison. Numerical results calculated by the present method are checked against available results published in the open literature to evaluate accuracy and validity. New results in terms of frequency parameters and mode shapes of CNTRC beams with elastic boundary conditions are presented here to provide a benchmark for future researchers in this field.

### Theory and formulation

**Figure 1:**
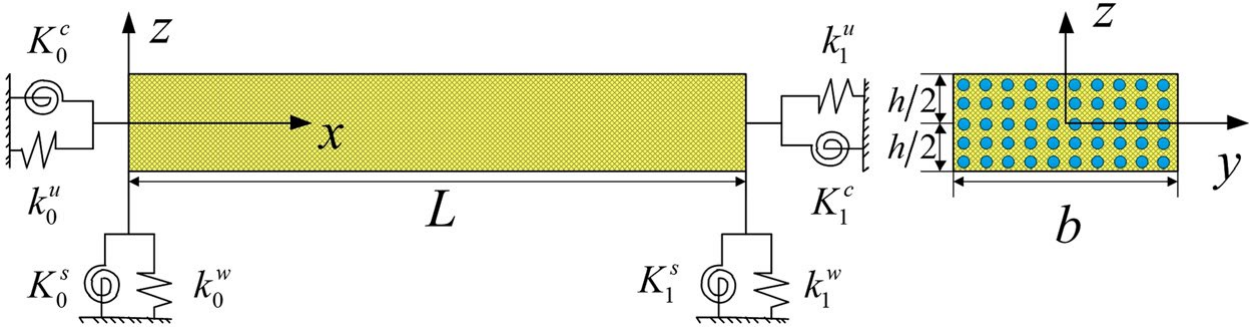
Geometry and coordinate system of a CNTRC beam. A right-handed Cartesian coordinate system is established in which the *x-*, *y-* and *z*-axes are taken along the length *L*, width *b* and height *h* of the beam, respectively. Four sets of springs (${k}_{0,L}^{u}$, ${k}_{0,L}^{w}$, ${K}_{0,L}^{s}$ and ${K}_{0,L}^{c}$) are artificially introduced to simulate boundary restraint forces at the two ends of the beam.

As depicted in Fig. 1, we consider a general CNT-reinforced composite beam with a rectangular cross section. A right-handed Cartesian coordinate system is established in which the length *L*, width *b*, and thickness *h* are respectively defined along the *x-*, *y-* and *z-*directions. The main purpose of this work is to investigate the CNTRC beam with arbitrary boundary conditions, and thus, two sets of linear springs (${k}_{0,L}^{u}$ and ${k}_{0,L}^{w}$) and two sets of rotational springs (${K}_{0,L}^{s}$ and ${K}_{0,L}^{c}$) are artificially introduced to simulate boundary restraint forces at the two ends of the beam. By assigning a suitable stiffness to the four sets of boundary springs, an arbitrary combination of classical and elastic boundary conditions can be realized. For example, if linear and rotational restraining spring coefficients at both ends are set to infinity (or a sufficiently large number in practical numerical simulations), the perfectly clamped boundary condition can be conveniently achieved.

**Figure 2:**
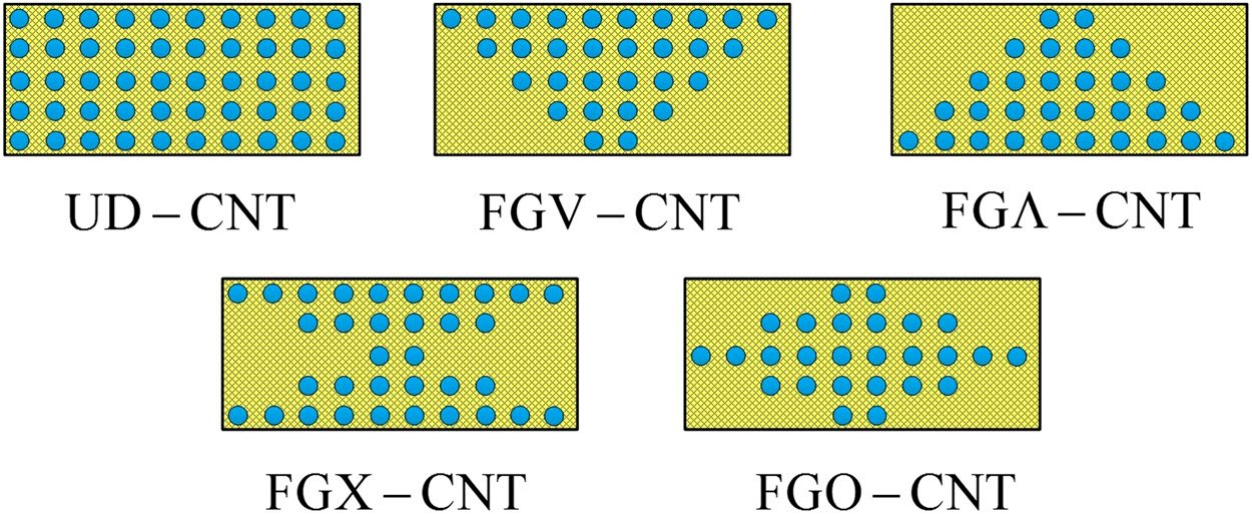
Cross section of UD, FG-Λ, FG-V, FG-O and FG-X CNTRC beams.

It is assumed that the CNTRC beam consists of a polymer matrix mixture that can be generally treated as an isotropic material and CNTs. SWCNT reinforcements are placed along the length direction and are either UD or FG in the thickness direction. Four- types of FG distribution forms are taken into account, namely, FG-X, FG-O, FG-V and FG-$\Lambda$, as illustrated in Fig. 2.

Regardless of the various distribution patterns of CNT reinforcement at the cross section, four types of FG-CNTRC beams are assumed to contain an equal CNT total weight of *m*_*tcnt*_ and total CNT volume fraction *V*_*tcnt*_. The expressions of CNT volume fraction for different distributions can be written as:

1a $${\rm{UD}}:\,{V}_{cnt}={V}_{tcnt}$$

1b $${\rm{FG}}\mbox{-}{\rm{X}}:\,{V}_{cnt}=4\frac{|z|}{h}{V}_{tcnt}$$

1c $${\rm{FG}}\mbox{-}{\rm{V}}:\,{V}_{cnt}=(1+\frac{2z}{h}){V}_{tcnt}$$

1d $${\rm{FG}}\mbox{-}{\rm{O}}:\,{V}_{cnt}=2-4\frac{|z|}{h}{V}_{tcnt}$$

1e $${\rm{FG}}\mbox{-}{\rm{\Lambda }}:\,{V}_{cnt}=(1-\frac{2z}{h}){V}_{tcnt}$$

The effective material properties of CNTRC beams, e.g., the Young’s modulus (*E*_11_ and *E*_22_), shear modulus (*G*_12_) and Poisson’s ratios (*v*_12_ and *v*_21_), are estimated according to matching molecular dynamics simulation results based on the rule of mixtures, which can be expressed as follows:

2a $${E}_{11}={\eta }_{1}{V}_{cnt}{E}_{11}^{cnt}+{V}_{m}{E}^{m}$$

2b $$\frac{{\eta }_{2}}{{E}_{22}}=\frac{{V}_{cnt}}{{E}_{22}^{cnt}}+\frac{{V}_{m}}{{E}^{m}}$$

2c $$\frac{{\eta }_{3}}{{G}_{12}}=\frac{{V}_{cnt}}{{G}_{12}^{cnt}}+\frac{{V}_{m}}{{G}^{m}}$$

2d $${V}_{m}=1-{V}_{cnt}$$

2e $${\upsilon }_{12}={V}_{cnt}{\upsilon }_{12}^{cnt}+{V}_{m}{\upsilon }^{m}$$

2f $${\upsilon }_{21}=\frac{{\upsilon }_{12}}{{E}_{11}}{E}_{22}$$

2g $$\rho ={V}_{cnt}{\rho }^{cnt}+{V}_{m}{\rho }^{m}$$

where ${E}_{11}^{cnt}$, ${E}_{22}^{cnt}$ and ${G}_{12}^{cnt}$ indicate the Young’s modulus along the longitudinal direction, Young’s modulus in the transverse direction and shear modulus of CNTs, respectively. *E* ^*m*^ and *G* ^*m*^ indicate the Young’s modulus and shear modulus of the isotropic matrix, respectively. ${\upsilon }_{12}^{cnt}$ and ${\rho }^{cnt}$ represent the Poisson’s ratio and mass density of CNT, respectively, and *v* ^*m*^ and ${\rho }^{m}$ are the corresponding properties of the matrix. *V*_*m*_ represents the matrix volume fraction, and *η*_*j*_ (*j* = 1, 2, 3) denotes the CNT efficiency parameters, which are determined from the results of molecular dynamics simulations.

The displacement field for the CNTRC beam under the assumptions of first-order shear deformation elasticity theory can be expressed as follows:

3a $$U(x,z,t)=u(x,t)+z\theta (x,t)$$

3b $$V(x,z,t)=z\varphi (x,t)$$

3c $$W(x,z,t)=w(x,t)$$

where *u* and *w* are the axial and transverse displacements along the *x-* and *z*-directions in the middle surface, respectively, and $\theta$ and $\varphi$ represent the rotations of the normal to the section about the *y-* and *x*-axes, respectively.

The strain and curvatures are defined in terms of the mid-plane displacements and rotations as:

4a $${\varepsilon }_{x}=\partial u/\partial x+z\partial \theta /\partial x$$

4b $${\gamma }_{xz}=\partial w/\partial x+\theta $$

4c $${\gamma }_{xy}=z\partial \varphi /\partial x$$

4d $${\varepsilon }_{x}^{0}=\partial u/\partial x$$

4e $${k}_{x}=\partial \theta /\partial x$$

4f $${k}_{xy}=\partial \varphi /\partial x$$

The constitutive equations are given by:

5a $$\{\begin{array}{c}{N}_{x}\\ {N}_{y}\\ {N}_{xy}\\ {M}_{x}\\ {M}_{y}\\ {M}_{xy}\end{array}\}=[\begin{array}{cccccc}{A}_{11} & {A}_{12} & {A}_{16} & {B}_{11} & {B}_{12} & {B}_{16}\\ {A}_{{\rm{12}}} & {A}_{22} & {A}_{26} & {B}_{{\rm{12}}} & {B}_{22} & {B}_{26}\\ {A}_{{\rm{16}}} & {A}_{{\rm{26}}} & {A}_{66} & {B}_{{\rm{16}}} & {B}_{{\rm{26}}} & {B}_{66}\\ {B}_{11} & {B}_{12} & {B}_{16} & {D}_{11} & {D}_{12} & {D}_{16}\\ {B}_{{\rm{12}}} & {B}_{22} & {B}_{26} & {D}_{{\rm{12}}} & {D}_{22} & {D}_{26}\\ {B}_{{\rm{16}}} & {B}_{{\rm{26}}} & {B}_{66} & {D}_{{\rm{16}}} & {D}_{{\rm{26}}} & {D}_{66}\end{array}]\{\begin{array}{c}{\varepsilon }_{x}^{0}\\ {\varepsilon }_{y}^{0}\\ {\gamma }_{xy}\\ {k}_{x}\\ {k}_{y}\\ {k}_{xy}\end{array}\}$$

5b $$\{\begin{array}{c}{Q}_{xz}\\ {Q}_{yz}\end{array}\}=[\begin{array}{cc}{A}_{55} & 0\\ 0 & {A}_{55}\end{array}]\{\begin{array}{c}{\gamma }_{xz}\\ {\gamma }_{yz}\end{array}\}$$

in which ${\varepsilon }_{x}$ and *γ*_*xz*_ denote the normal and shear strain, respectively. ${\varepsilon }_{x}^{0}$, ${\varepsilon }_{y}^{0}$ and *γ*_*xy*_ represent the strain at the middle surface; *k*_*x*_, *k*_*y*_ and *k*_*xy*_ are the bending and twisting curvatures; *N*_*x*_, *N*_*y*_ and *N*_*xy*_ indicate the force resultants at the middle surface; *M*_*x*_, *M*_*y*_ and *M*_*xy*_ are the bending and twisting moment resultants; and *Q*_*xy*_ and *Q*_*yz*_ represent the shear force resultants.

Regarding the CNTRC beam, certain force and moment resultants, namely, *N*_*y*_, *N*_*xy*_, *Q*_*yz*_ and *M*_*y*_, are equal to zero, while the corresponding strains ${\varepsilon }_{y}^{0}$, *γ*_*xy*_ and curvature *k*_*y*_ are assumed to be non-zero. Consequently, Eq. (5) can be expressed as:

6a $$\{\begin{array}{c}{N}_{x}\\ {M}_{x}\\ {M}_{xy}\end{array}\}=[\begin{array}{ccc}{\bar{A}}_{11} & {\bar{B}}_{11} & {\bar{B}}_{16}\\ {\bar{B}}_{11} & {\bar{D}}_{11} & {\bar{D}}_{16}\\ {\bar{B}}_{16} & {\bar{D}}_{16} & {\bar{D}}_{66}\end{array}]\{\begin{array}{c}\partial u/\partial x\\ \partial \theta /\partial x\\ \partial \varphi /\partial x\end{array}\}$$

6b $${Q}_{xz}={A}_{55}{\gamma }_{xz}={A}_{55}(\partial w/\partial x+\theta )$$

where

7 $$\begin{array}{c}[\begin{array}{ccc}{\bar{A}}_{11} & {\bar{B}}_{11} & {\bar{B}}_{16}\\ {\bar{B}}_{11} & {\bar{D}}_{11} & {\bar{D}}_{16}\\ {\bar{B}}_{16} & {\bar{D}}_{16} & {\bar{D}}_{66}\end{array}]=[\begin{array}{ccc}{A}_{11} & {B}_{11} & {B}_{16}\\ {B}_{11} & {D}_{11} & {D}_{16}\\ {B}_{16} & {D}_{16} & {D}_{66}\end{array}]-\\ \begin{array}{l}\end{array}[\begin{array}{ccc}{A}_{12} & {A}_{16} & {B}_{12}\\ {B}_{12} & {B}_{16} & {D}_{12}\\ {B}_{26} & {D}_{12} & {D}_{26}\end{array}]\,{[\begin{array}{ccc}{A}_{22} & {A}_{26} & {B}_{22}\\ {A}_{26} & {A}_{66} & {B}_{26}\\ {B}_{22} & {B}_{26} & {D}_{22}\end{array}]}^{-1}{[\begin{array}{ccc}{A}_{12} & {A}_{16} & {B}_{12}\\ {B}_{12} & {B}_{16} & {D}_{12}\\ {B}_{26} & {D}_{12} & {D}_{26}\end{array}]}^{T}\end{array}$$

The extensional stiffness coefficients *A*_*ij*_, coupling stiffness coefficients *B*_*ij*_, bending stiffness coefficients *D*_*ij*_(*i, j* = 1, 2, 6) and transverse shear stiffness *A*_55_ are defined as functions of material properties, which can be written as:

8a $$({A}_{ij},{B}_{ij},{D}_{ij})={\int }_{-h/2}^{h/2}{\bar{Q}}_{ij}(1,z,{z}^{2})dz$$

8b $${A}_{55}=\kappa {\int }_{-h/2}^{h/2}{\bar{Q}}_{55}dz$$

where $\kappa$ denotes the shear correction factor. The reduced stiffness coefficients ${\bar{Q}}_{ij}(i=1,2,6)$ are defined by the following equations:

9a $${Q}_{11}=\frac{{E}_{11}}{1-{\upsilon }_{12}{\upsilon }_{21}}$$

9b $${Q}_{12}=\frac{{\upsilon }_{12}{E}_{22}}{1-{\upsilon }_{12}{\upsilon }_{21}}=\frac{{\upsilon }_{21}{E}_{11}}{1-{\upsilon }_{12}{\upsilon }_{21}}$$

9c $${Q}_{22}=\frac{{E}_{22}}{1-{\upsilon }_{12}{\upsilon }_{21}}$$

9d $${Q}_{66}={Q}_{55}={G}_{12}$$

9e $${Q}_{16}={Q}_{26}=0$$

With the aim of deriving the governing equations and boundary conditions of the CNTRC beam according to Hamilton’s principle, the energy expressions are defined as follows. The total linear elastic strain energy (*U*_*s*_) function of the CNTRC beam can be expressed as

10 $$\begin{array}{rcl}{U}_{s} & = & \frac{b}{2}{\int }_{0}^{L}({N}_{x}{\varepsilon }_{x}^{0}+{M}_{x}{k}_{x}+{M}_{xy}{k}_{xy}+{Q}_{xz}{\gamma }_{xz})dx\\  & = & \frac{b}{2}{\int }_{0}^{L}\{\begin{array}{c}{\bar{A}}_{11}{({\varepsilon }_{x}^{0})}^{2}+2{\bar{B}}_{11}{\varepsilon }_{x}^{0}{k}_{x}+2{\bar{B}}_{16}{\varepsilon }_{x}^{0}{k}_{xy}+{\bar{D}}_{11}{({k}_{x})}^{2}\\ +2{\bar{D}}_{16}{k}_{x}{k}_{xy}+{\bar{D}}_{66}{({k}_{xy})}^{2}+{A}_{55}{({\gamma }_{xz})}^{2}\end{array}\}dx\end{array}$$

and the homologous kinetic energy (*T*) function is given by

11 $$\begin{array}{c}T=\,\frac{b}{2}{\int }_{0}^{L}{\int }_{-h/2}^{h/2}\rho ({\dot{u}}^{2}+{\dot{v}}^{2}+{\dot{w}}^{2})dzdx\\ \,\,\,=\,\frac{b}{2}{\int }_{0}^{L}[{I}_{1}{({\dot{u}}_{0})}^{2}+{I}_{3}{(\dot{\theta })}^{2}+{I}_{3}{(\mathop{\varphi }\limits^{.})}^{2}+2{I}_{2}{\dot{u}}_{0}\dot{\theta }+{I}_{1}({\dot{w}}_{0})]dx\end{array}$$

in which the inertia terms can be written as

12 $$({I}_{1},{I}_{2},{I}_{3})={\int }_{-h/2}^{h/2}\rho (1,z,{z}^{2})dz$$

In addition, four sets of boundary springs are introduced at each end of the beam; the boundary springs deformation strain energy (*U*_*sp*_) function is given by

13 $${U}_{sp}=\frac{1}{2}{[{k}_{0}^{u}{u}^{2}+{k}_{0}^{w}{w}^{2}+{K}_{0}^{c}{\theta }^{2}+{K}_{0}^{s}{\varphi }^{2}]}_{x=0}+\frac{1}{2}{[{k}_{L}^{u}{u}^{2}+{k}_{L}^{w}{w}^{2}+{K}_{L}^{c}{\theta }^{2}+{K}_{L}^{s}{\varphi }^{2}]}_{x=L}$$

and the Hamilton’s principle with regard to the arbitrary initial time *t*_1_ and final time *t*_2_ is given by

14 $$\delta {\int }_{{t}_{1}}^{{t}_{2}}(T-{U}_{s}-{U}_{sp})dt=0$$

Substituting Eqs (10), (11) and (13) into Eq. (14) and integrating by parts to eliminate the variational terms, the governing equations and boundary conditions can be obtained as follows:

15 $$\begin{array}{c}{\int }_{{t}_{1}}^{{t}_{2}}{\int }_{0}^{L}({\bar{A}}_{11}\frac{{\partial }^{2}u}{\partial {x}^{{\rm{2}}}}+{\bar{B}}_{11}\frac{{\partial }^{2}\theta }{\partial {x}^{{\rm{2}}}}+{\bar{B}}_{{\rm{16}}}\frac{{\partial }^{2}\varphi }{\partial {x}^{{\rm{2}}}}-{I}_{1}\frac{{\partial }^{2}u}{\partial {t}^{{\rm{2}}}}-{I}_{2}\frac{{\partial }^{2}\theta }{\partial {t}^{{\rm{2}}}})\delta udxdt\\ +{\int }_{{t}_{1}}^{{t}_{2}}{\int }_{0}^{L}({A}_{55}\frac{{\partial }^{2}w}{\partial {x}^{{\rm{2}}}}+{A}_{55}\frac{\partial \theta }{\partial x}-{I}_{1}\frac{{\partial }^{2}w}{\partial {t}^{{\rm{2}}}})\delta wdxdt\\ +{\int }_{{t}_{1}}^{{t}_{2}}{\int }_{0}^{L}({\bar{B}}_{11}\frac{{\partial }^{2}u}{\partial {x}^{{\rm{2}}}}+{\bar{D}}_{11}\frac{{\partial }^{2}\theta }{\partial {x}^{{\rm{2}}}}+{\bar{D}}_{{\rm{16}}}\frac{{\partial }^{2}\varphi }{\partial {x}^{{\rm{2}}}}-{A}_{55}\frac{\partial w}{\partial x}-{A}_{55}\theta -{I}_{2}\frac{{\partial }^{2}u}{\partial {t}^{{\rm{2}}}}-{I}_{3}\frac{{\partial }^{2}\theta }{\partial {t}^{{\rm{2}}}})\delta \theta dxdt\\ +{\int }_{{t}_{1}}^{{t}_{2}}{\int }_{0}^{L}({\bar{B}}_{16}\frac{{\partial }^{2}u}{\partial {x}^{{\rm{2}}}}+{\bar{D}}_{16}\frac{{\partial }^{2}\theta }{\partial {x}^{{\rm{2}}}}+{\bar{D}}_{{\rm{66}}}\frac{{\partial }^{2}\varphi }{\partial {x}^{{\rm{2}}}}-{I}_{3}\frac{{\partial }^{2}\varphi }{\partial {t}^{{\rm{2}}}})\delta \varphi dxdt-\\ {\int }_{{t}_{1}}^{{t}_{2}}[\begin{array}{c}({N}_{x}+{k}_{L}^{u}u){\delta u|}_{L}-({N}_{x}-{k}_{0}^{u}u){\delta u|}_{0}+({Q}_{xz}+{k}_{L}^{w}w){\delta w|}_{L}-({Q}_{xz}-{k}_{0}^{w}w){\delta w|}_{0}\\ +({M}_{x}+{K}_{L}^{c}\theta ){\delta \theta |}_{L}-({M}_{x}-{K}_{0}^{c}\theta ){\delta \theta |}_{0}+({M}_{xy}+{K}_{L}^{s}\varphi ){\delta \varphi |}_{L}-({M}_{xy}-{K}_{0}^{s}\varphi ){\delta \varphi |}_{0}\end{array}]=0\end{array}$$

Because of the arbitrariness of the virtual displacements *δu* and *δw*, only when the values of the virtual displacements coefficients are equal to zero is Eq. (15) tractable. The governing equations can be expressed in terms of the differentials of displacement components as

16a $${\bar{A}}_{11}\frac{{\partial }^{2}u}{\partial {x}^{{\rm{2}}}}+{\bar{B}}_{11}\frac{{\partial }^{2}\theta }{\partial {x}^{{\rm{2}}}}+{\bar{B}}_{{\rm{16}}}\frac{{\partial }^{2}\varphi }{\partial {x}^{{\rm{2}}}}={I}_{1}\frac{{\partial }^{2}u}{\partial {t}^{{\rm{2}}}}+{I}_{2}\frac{{\partial }^{2}\theta }{\partial {t}^{{\rm{2}}}}$$

16b $${A}_{55}\frac{{\partial }^{2}w}{\partial {x}^{{\rm{2}}}}+{A}_{55}\frac{\partial \theta }{\partial x}={I}_{1}\frac{{\partial }^{2}w}{\partial {t}^{{\rm{2}}}}$$

16c $${\bar{B}}_{11}\frac{{\partial }^{2}u}{\partial {x}^{{\rm{2}}}}+{\bar{D}}_{11}\frac{{\partial }^{2}\theta }{\partial {x}^{{\rm{2}}}}+{\bar{D}}_{{\rm{16}}}\frac{{\partial }^{2}\varphi }{\partial {x}^{{\rm{2}}}}-{A}_{55}\frac{\partial w}{\partial x}-{A}_{55}\theta ={I}_{2}\frac{{\partial }^{2}u}{\partial {t}^{{\rm{2}}}}+{I}_{3}\frac{{\partial }^{2}\theta }{\partial {t}^{{\rm{2}}}}$$

16d $${\bar{B}}_{16}\frac{{\partial }^{2}u}{\partial {x}^{{\rm{2}}}}+{\bar{D}}_{16}\frac{{\partial }^{2}\theta }{\partial {x}^{{\rm{2}}}}+{\bar{D}}_{{\rm{66}}}\frac{{\partial }^{2}\varphi }{\partial {x}^{{\rm{2}}}}={I}_{3}\frac{{\partial }^{2}\varphi }{\partial {t}^{{\rm{2}}}}$$

and the general boundary conditions can be stated as

17a $$x=0:\{\begin{array}{c}{N}_{x}-{k}_{0}^{u}u=0\\ {Q}_{xz}-{k}_{0}^{w}w=0\\ {M}_{x}-{K}_{0}^{c}\theta =0\\ {M}_{xy}-{K}_{0}^{s}\varphi =0\end{array}$$

17b $$x=L:\{\begin{array}{c}{N}_{x}+{k}_{L}^{u}u=0\\ {Q}_{xz}+{k}_{L}^{w}w=0\\ {M}_{x}+{K}_{L}^{c}\theta =0\\ {M}_{xy}+{K}_{L}^{s}\varphi =0\end{array}$$

Therefore, all classical boundary conditions and elastic supports can be directly achieved by means of the artificial spring boundary technique to assign the rigidities of the boundary springs at a certain value.

The appropriate choice of admissible displacement functions plays a significant role to ensure the validity and accuracy of the proposed solution procedures. Eqs (16a–16d) indicate that the translation and rotations displacements of the CNTRC beam are required up to the second derivative. For the sake of satisfying the arbitrary boundary conditions at both ends of the beam, the displacements and rotational components are represented as 1D Fourier cosine series expansions with two supplemental auxiliary function terms. These terms are introduced to improve the convergence of the primary Fourier series representations and avoid the potential discontinuities of the displacement functions and their first-order derivatives at the boundaries. Accordingly, the functions of flexural displacements and rotation of the CNTRC beam can be universally expressed as

18a $$u(x)=\sum _{m=0}^{M}{A}_{m}\,\cos \,{\lambda }_{m}x+{a}_{1}{P}_{1}(x)+{a}_{2}{P}_{2}(x)$$

18b $$w(x)=\sum _{m=0}^{M}{B}_{m}\,\cos \,{\lambda }_{m}x+{b}_{1}{P}_{1}(x)+{b}_{2}{P}_{2}(x)$$

18c $$\theta (x)=\sum _{m=0}^{M}{C}_{m}\,\cos \,{\lambda }_{m}x+{c}_{1}{P}_{1}(x)+{c}_{2}{P}_{2}(x)$$

18d $$\varphi (x)=\sum _{m=0}^{M}{D}_{m}\,\cos \,{\lambda }_{m}x+{d}_{1}{P}_{1}(x)+{d}_{2}{P}_{2}(x)$$

where ${\lambda }_{m}=m\pi /L$. *M* represents the truncation number, *A*_m_, *B*_m_, *C*_m_ and *D*_m_ are the expansion coefficients of the standard Fourier series, and *a*_*i*_, *b*_*i*_ and *c*_*i*_ (*i* = 1, 2) denote the corresponding expansion coefficients of the auxiliary function *P*_1_(*x*) and *P*_2_(*x*), which are defined as:

19a $${P}_{1}(x)=x{(\frac{x}{L}-1)}^{2}$$

19b $${P}_{2}(x)=\frac{{x}^{2}}{L}(\frac{x}{L}-1)$$

According to the modified Fourier series, the free-vibration characteristics of the CNTRC beam can be solved by means of strong-form solution procedures and weak-form solutions, as described below. The strong-form solution procedure is given step by step as follows.

We rewrite Eq. (18) in matrix form as

20 $$\begin{array}{c}u(x)={{\bf{H}}}_{f}{\bf{A}}+{{\bf{H}}}_{a}{\bf{a}}\\ w(x)={{\bf{H}}}_{f}{\bf{B}}+{{\bf{H}}}_{a}{\bf{b}}\\ \theta (x)={{\bf{H}}}_{f}{\bf{C}}+{{\bf{H}}}_{a}{\bf{c}}\\ \varphi (x)={{\bf{H}}}_{f}{\bf{D}}+{{\bf{H}}}_{a}{\bf{d}}\end{array}$$

where

21a $${{\bf{H}}}_{f}=[\begin{array}{ccccc}\cos \,{\lambda }_{0}x, & \cdots  & \cos \,{\lambda }_{m}x, & \cdots  & \cos \,{\lambda }_{M}x\end{array}]$$

21b $${{\bf{H}}}_{a}=[{P}_{1}(x),{P}_{2}(x)]$$

21c $$\begin{array}{cc}{\bf{A}}={[\begin{array}{ccccc}{A}_{0}, & \cdots  & ,{A}_{m} & \cdots  & ,{A}_{M}\end{array}]}^{T} & {\bf{a}}={[{a}_{1},{a}_{2}]}^{T}\end{array}$$

21d $$\begin{array}{cc}{\bf{B}}={[\begin{array}{ccccc}{B}_{0}, & \cdots  & ,{B}_{m} & \cdots  & ,{B}_{M}\end{array}]}^{T} & {\bf{b}}={[{b}_{1},{b}_{2}]}^{T}\end{array}$$

21e $$\begin{array}{cc}{\bf{C}}={[\begin{array}{ccccc}{C}_{0}, & \cdots  & ,{C}_{m} & \cdots  & ,{C}_{M}\end{array}]}^{T} & {\bf{c}}={[{c}_{1},{c}_{2}]}^{T}\end{array}$$

21f $$\begin{array}{cc}{\bf{D}}={[\begin{array}{ccccc}{D}_{0}, & \cdots  & ,{D}_{m} & \cdots  & ,{D}_{M}\end{array}]}^{T} & {\bf{d}}={[{d}_{1},{d}_{2}]}^{T}\end{array}$$

Substituting Eqs (20) and (21) into Eq. (16), we obtain

22 $${{\bf{L}}}_{f}\,[\begin{array}{c}{\bf{A}}\\ {\bf{B}}\\ {\bf{C}}\\ {\bf{D}}\end{array}]+{{\bf{L}}}_{a}\,[\begin{array}{c}{\bf{a}}\\ {\bf{b}}\\ {\bf{c}}\\ {\bf{d}}\end{array}]-{\omega }^{2}\,({{\bf{M}}}_{f}\,[\begin{array}{c}{\bf{A}}\\ {\bf{B}}\\ {\bf{C}}\\ {\bf{D}}\end{array}]+{{\bf{M}}}_{a}\,[\begin{array}{c}{\bf{a}}\\ {\bf{b}}\\ {\bf{c}}\\ {\bf{d}}\end{array}])=0$$

in which

23 $$\begin{array}{c}{{\bf{L}}}_{i}=[\begin{array}{cccc}{L}_{11}{{\bf{H}}}_{i} & 0 & {L}_{13}{{\bf{H}}}_{i} & {L}_{14}{{\bf{H}}}_{i}\\ 0 & {L}_{22}{{\bf{H}}}_{i} & {L}_{23}{{\bf{H}}}_{i} & 0\\ {L}_{31}{{\bf{H}}}_{i} & {L}_{32}{{\bf{H}}}_{i} & {L}_{33}{{\bf{H}}}_{i} & {L}_{34}{{\bf{H}}}_{i}\\ {L}_{41}{{\bf{H}}}_{i} & 0 & {L}_{43}{{\bf{H}}}_{i} & {L}_{44}{{\bf{H}}}_{i}\end{array}](i=f,a)\\ {{\bf{M}}}_{i}=[\begin{array}{cccc}{M}_{11}{{\bf{H}}}_{i} & 0 & {M}_{13}{{\bf{H}}}_{i} & 0\\ 0 & {M}_{22}{{\bf{H}}}_{i} & 0 & 0\\ {M}_{31}{{\bf{H}}}_{i} & 0 & {M}_{33}{{\bf{H}}}_{i} & 0\\ 0 & 0 & 0 & {M}_{44}{{\bf{H}}}_{i}\end{array}](i=f,a)\end{array}$$

and the coefficients of the linear operator are defined as follows

24 $$\begin{array}{l}{L}_{11}={\bar{A}}_{11}\frac{{\partial }^{2}}{\partial {x}^{{\rm{2}}}},{L}_{13}={\bar{B}}_{11}\frac{{\partial }^{2}}{\partial {x}^{{\rm{2}}}},{L}_{14}={\bar{B}}_{{\rm{16}}}\frac{{\partial }^{2}}{\partial {x}^{{\rm{2}}}},\,{L}_{22}={A}_{55}\frac{{\partial }^{2}}{\partial {x}^{{\rm{2}}}},{L}_{23}={A}_{55}\frac{\partial }{\partial x}\\ {L}_{31}={\bar{B}}_{11}\frac{{\partial }^{2}}{\partial {x}^{{\rm{2}}}},{L}_{32}=-{A}_{55}\frac{\partial }{\partial x},{L}_{33}={\bar{D}}_{11}\frac{{\partial }^{2}}{\partial {x}^{{\rm{2}}}}-{A}_{55},{L}_{34}={\bar{D}}_{{\rm{16}}}\frac{{\partial }^{2}}{\partial {x}^{{\rm{2}}}}\\ {L}_{41}={\bar{B}}_{16}\frac{{\partial }^{2}}{\partial {x}^{{\rm{2}}}},{L}_{43}={\bar{D}}_{16}\frac{{\partial }^{2}}{\partial {x}^{{\rm{2}}}},{L}_{44}={\bar{D}}_{{\rm{66}}}\frac{{\partial }^{2}}{\partial {x}^{{\rm{2}}}}\\ {M}_{11}={M}_{22}=-{I}_{1},{M}_{13}={M}_{31}=-{I}_{2},{M}_{33}={M}_{44}=-{I}_{3}\end{array}$$

Similarly, substituting Eqs (20) and (21) into Eq. (17), the boundary conditions of CNTRC beams are stated as

25 $$[\begin{array}{c}{{\bf{L}}}_{f}^{bc0}\\ {{\bf{L}}}_{f}^{bcL}\end{array}]\,[\begin{array}{c}{\bf{A}}\\ {\bf{B}}\\ {\bf{C}}\\ {\bf{D}}\end{array}]+[\begin{array}{c}{{\bf{L}}}_{a}^{bc0}\\ {{\bf{L}}}_{a}^{bcL}\end{array}]\,[\begin{array}{c}{\bf{a}}\\ {\bf{b}}\\ {\bf{c}}\\ {\bf{d}}\end{array}]=0$$

where

26a $${{\bf{L}}}_{i}^{bc0}={[\begin{array}{cccc}{\bar{A}}_{11}\frac{\partial {{\bf{H}}}_{i}}{\partial x}-{k}_{0}^{u}{{\bf{H}}}_{i} & 0 & {\bar{B}}_{11}\frac{\partial {{\bf{H}}}_{i}}{\partial x} & {\bar{B}}_{16}\frac{\partial {{\bf{H}}}_{i}}{\partial x}\\ 0 & {A}_{55}\frac{\partial {{\bf{H}}}_{i}}{\partial x}-{k}_{0}^{w}{{\bf{H}}}_{i} & {A}_{55}{{\bf{H}}}_{i} & 0\\ {\bar{B}}_{11}\frac{\partial {{\bf{H}}}_{i}}{\partial x} & 0 & {\bar{D}}_{11}\frac{\partial {{\bf{H}}}_{i}}{\partial x}-{K}_{0}^{c}{{\bf{H}}}_{i} & {\bar{D}}_{16}\frac{\partial {{\bf{H}}}_{i}}{\partial x}\\ {\bar{B}}_{16}\frac{\partial {{\bf{H}}}_{i}}{\partial x} & 0 & {\bar{D}}_{16}\frac{\partial {{\bf{H}}}_{i}}{\partial x} & {\bar{D}}_{66}\frac{\partial {{\bf{H}}}_{i}}{\partial x}-{K}_{0}^{s}{{\bf{H}}}_{i}\end{array}]}_{x=0}(i=f,a)$$

26b $${{\bf{L}}}_{i}^{bcL}={[\begin{array}{cccc}{\bar{A}}_{11}\frac{\partial {{\bf{H}}}_{i}}{\partial x}+{k}_{L}^{u}{{\bf{H}}}_{i} & 0 & {\bar{B}}_{11}\frac{\partial {{\bf{H}}}_{i}}{\partial x} & {\bar{B}}_{16}\frac{\partial {{\bf{H}}}_{i}}{\partial x}\\ 0 & {A}_{55}\frac{\partial {{\bf{H}}}_{i}}{\partial x}+{k}_{L}^{w}{{\bf{H}}}_{i} & {A}_{55}{{\bf{H}}}_{i} & 0\\ {\bar{B}}_{11}\frac{\partial {{\bf{H}}}_{i}}{\partial x} & 0 & {\bar{D}}_{11}\frac{\partial {{\bf{H}}}_{i}}{\partial x}+{K}_{L}^{c}{{\bf{H}}}_{i} & {\bar{D}}_{16}\frac{\partial {{\bf{H}}}_{i}}{\partial x}\\ {\bar{B}}_{16}\frac{\partial {{\bf{H}}}_{i}}{\partial x} & 0 & {\bar{D}}_{16}\frac{\partial {{\bf{H}}}_{i}}{\partial x} & {\bar{D}}_{66}\frac{\partial {{\bf{H}}}_{i}}{\partial x}+{K}_{L}^{s}{{\bf{H}}}_{i}\end{array}]}_{x=L}(i=f,a)$$

Therefore, the expansion coefficients of the standard Fourier cosine series and corresponding auxiliary functions have a certain relationship according to the boundary conditions, which are expressed as:

27 $$[\begin{array}{c}{\bf{a}}\\ {\bf{b}}\\ {\bf{c}}\\ {\bf{d}}\end{array}]=-{[\begin{array}{c}{{\bf{L}}}_{a}^{bc0}\\ {{\bf{L}}}_{a}^{bcL}\end{array}]}^{-1}\,[\begin{array}{c}{{\bf{L}}}_{f}^{bc0}\\ {{\bf{L}}}_{f}^{bcL}\end{array}]\,[\begin{array}{c}{\bf{A}}\\ {\bf{B}}\\ {\bf{C}}\\ {\bf{D}}\end{array}]$$

Substituting Eqs (26) and (27) into Eq. (22), then multiplying the transpose of displacement functions matrix **H**_*f*_ on the left side and integrating both sides of the equality from 0 to *L* with respect to *x*, the partial differential equations are transformed into a standard eigenvalue problem as follows:

28 $$({\bf{K}}-{\omega }^{2}{\bf{M}}){\bf{G}}=0$$

in which **K** and **M** are the stiffness matrix and mass matrix, respectively, **G** is a vector that contains all undetermined coefficients of the standard Fourier series, and these matrices can be written as:

29a $${\bf{K}}={{\bf{L}}}_{f}-{{\bf{L}}}_{a}{[\begin{array}{c}{{\bf{L}}}_{a}^{bc0}\\ {{\bf{L}}}_{a}^{bcL}\end{array}]}^{-1}\,[\begin{array}{c}{{\bf{L}}}_{f}^{bc0}\\ {{\bf{L}}}_{f}^{bcL}\end{array}]$$

29b $${\bf{M}}={{\bf{M}}}_{f}-{{\bf{M}}}_{a}{[\begin{array}{c}{{\bf{L}}}_{a}^{bc0}\\ {{\bf{L}}}_{a}^{bcL}\end{array}]}^{-1}\,[\begin{array}{c}{{\bf{L}}}_{f}^{bc0}\\ {{\bf{L}}}_{f}^{bcL}\end{array}]$$

29c $${\bf{G}}={[\begin{array}{cccc}{\bf{A}} & {\bf{B}} & {\bf{C}} & {\bf{D}}\end{array}]}^{T}$$

The natural frequencies and modes of CNTRC beams can be obtained directly by solving the standard eigenvalue equation.

Exact solutions are often unavailable in complex vibration problems, and an approximate method is employed to complete the vibrational analysis. The Rayleigh–Ritz method associated with modified Fourier series, i.e., weak-form solution, is also presented below to compare with the strong-form solution.

In the Ritz-variational energy procedure, the accuracy of the solution will rest on how well the actual displacement can be faithfully represented by an appropriate admissible displacement field in general. Hence, auxiliary functions play a crucial role. The same displacement functions are selected for comparative purposes, and all expansion coefficients of the modified Fourier series can be regarded as generalized coordinates independently and equally.

With regard to free-vibration analysis, the Lagrange energy function of CNTRC beams consists of the strain energy, kinetic energy and boundary spring deformation strain energy as follows:

30 $$L=T-{U}_{s}-{U}_{sp}$$

Substituting Eqs (10), (11) and (13) into Eq. (30), minimizing the total expression of the Lagrange energy function via taking the derivatives of the equation with respect to the generalized coordinates and setting all expressions equal to zero to find the stationary value of the energy function, we obtain:

31 $$\frac{\partial L}{\partial {A}_{m}}=\frac{\partial L}{\partial {B}_{m}}=\frac{\partial L}{\partial {C}_{m}}=\frac{\partial L}{\partial {D}_{m}}=\frac{\partial L}{\partial {a}_{i}}=\frac{\partial L}{\partial {b}_{i}}=\frac{\partial L}{\partial {c}_{i}}=\frac{\partial L}{\partial {d}_{i}}=0,(i=1,2)$$

A total of 4(*M *+ 3) linear algebraic equations for the undetermined coefficients are achieved, which can be added and represented in a matrix form as

32 $$({{\bf{K}}}^{\ast }-{\omega }^{2}{{\bf{M}}}^{\ast }){{\bf{G}}}^{\ast }=0$$

where **G** ^*^ indicates the undetermined coefficients column vector, **K** ^*^ is treated as the total stiffness matrix of the CNTRC beams and **M** ^*^ indicates the corresponding mass matrix. Their expressions can be written as:

33a $${{\bf{G}}}^{\ast }={[\begin{array}{c}\begin{array}{ccccc}{A}_{0}, & \cdots  & ,{A}_{m} & \cdots  & ,{A}_{M}\end{array}\begin{array}{ccccc},{a}_{1},{a}_{2},{B}_{0}, & \cdots  & ,{B}_{m} & \cdots  & ,{B}_{M},\end{array}{b}_{1},{b}_{2},\\ \begin{array}{ccccc}{C}_{0}, & \cdots  & ,{C}_{m} & \cdots  & ,{C}_{M}\end{array},{c}_{1},{c}_{2},\begin{array}{ccccc}{D}_{0}, & \cdots  & ,{D}_{m} & \cdots  & ,{D}_{M}\end{array},{d}_{1},{d}_{2}\end{array}]}^{T}$$

33b $${{\bf{K}}}^{\ast }=[\begin{array}{cccc}{{\bf{K}}}_{uu} & 0 & {{\bf{K}}}_{u\theta } & {{\bf{K}}}_{u\varphi }\\ 0 & {{\bf{K}}}_{ww} & {{\bf{K}}}_{w\theta } & 0\\ {{\bf{K}}}_{u\theta }^{T} & {{\bf{K}}}_{w\theta }^{T} & {{\bf{K}}}_{\theta \theta } & {{\bf{K}}}_{\theta \varphi }\\ {{\bf{K}}}_{u\varphi }^{T} & 0 & {{\bf{K}}}_{\theta \varphi }^{T} & {{\bf{K}}}_{\varphi \varphi }\end{array}]$$

33c $${{\bf{M}}}^{\ast }=[\begin{array}{cccc}{{\bf{M}}}_{uu} & 0 & {{\bf{M}}}_{u\theta } & 0\\ 0 & {{\bf{M}}}_{ww} & 0 & 0\\ {{\bf{M}}}_{u\theta }^{T} & 0 & {{\bf{M}}}_{\theta \theta } & 0\\ 0 & 0 & 0 & {{\bf{M}}}_{\varphi \varphi }\end{array}]$$

The detailed expressions of the elements of **K** ^*^ and **M** ^*^ are listed in the Appendix. The Rayleigh–Ritz method associated with the modified Fourier series is equivalent to strong-form solution procedures to obtain the vibration results by solving a standard eigenvalue equation.

### Numerical results and discussions

With the achievement of the theoretical formulation of the modified Fourier method mentioned above, selected numerical examples for the free-vibration analysis of CNTRC beams with arbitrary boundary conditions are presented to validate the feasibility, accuracy and efficiency of the proposed method. Several key parameters representing the vibrational characteristics of CNTRC beams, such as the *L/h* ratio, CNT volume fraction and boundary spring stiffness, are discussed, and new results and useful conclusions are obtained.

The following material properties for CNTs and PMMA matrixes are applied unless otherwise illustrated: *E* ^*m*^ = 2.5 GPa, *v* ^*m*^=0.3, *ρ* ^*m*^ = 1150 kg/m^3^, ${E}_{11}^{cnt}=5645.6$ GPa, ${E}_{22}^{cnt}=7080$ GPa, ${G}_{12}^{cnt}=1944.5$ GPa, ${\upsilon }_{12}^{cnt}=0.175$ and ${\rho }^{cnt}=2100$ kg/m^3^. By matching with the results calculated from molecular dynamics simulations, three types of CNT efficiency parameters with special CNT volume fractions are given as: *η*_1_ = 0.137, *η*_2_ = 1.022 for *V*_*tcnt*_ = 0.12; *η*_1_ = 0.142, *η*_2_ = 1.138 for *V*_*tcnt*_ = 0.17; *η*_1_ = 0.141, *η*_2_ = 1.109 for *V*_*tcnt*_ = 0.28. In the absence of shear modulus results in molecular dynamics simulations, *η*_3_ is defined as 0.7 *η*_2_.

In addition, the non-dimensional frequency parameters of the natural frequency take the form of $\Omega =\omega {L}^{2}\sqrt{{\rho }^{m}/({E}^{m}{h}^{2})}$ in the latter subsections unless otherwise stated. The corresponding boundary conditions at the ends of beam can be defined in terms of the spring stiffness as:

**Clamped (C):** $${k}_{0,L}^{u}={k}_{0,L}^{w}={K}_{0,L}^{c}={K}_{0,L}^{s}={10}^{15}$$

**Simply supported (S):** $${k}_{0,L}^{u}={k}_{0,L}^{w}={K}_{0,L}^{s}={10}^{15},{K}_{0,L}^{c}=0$$

**Free (F):** $${k}_{0,L}^{u}={k}_{0,L}^{w}={K}_{0,L}^{c}={K}_{0,L}^{s}=0$$

**Elastically restrained case 1 (E1):** ${k}_{0,L}^{u}={K}_{0,L}^{c}={K}_{0,L}^{s}=0,{k}_{0,L}^{w}={10}^{8}$

**Elastically restrained case 2 (E2):** ${k}_{0,L}^{u}={K}_{0,L}^{s}=0,{k}_{0,L}^{w}={K}_{0,L}^{c}={10}^{8}$

The rationality of these definitions of boundary conditions in terms of assigning spring stiffness will be established through numerical examples in subsequent studies. For brevity, symbolism is applied to illustrate the boundary condition of FG-CNTRC beams, e.g., SE1 indicates a beam with S (simply supported) and E1 (elastically restrained case 1) boundary conditions at = 0 and *x* = *L*, respectively.

### Convergence and validation

As previously mentioned, modified Fourier series with infinite terms in the current solution framework are infinitely approximate in the real results. Nevertheless, the infinite terms must be numerically truncated in practical numerical simulations. Consequently, convergence studies are conducted to determine the number of series terms *M* used in the computation. The first four lowest frequency parameters Ω for perfectly clamped and simply supported FGV-CNT beams are considered in Table 1, in which the results obtained from strong-form solution procedures and the Rayleigh–Ritz method are given. Excellent convergence and satisfactory numerical stability of two types of current solutions can be observed. The frequency parameters Ω converge sharply as the number of series terms *M* increases from 4 to 15, and the results are almost invariant when the truncated number reaches a certain value (*M* = 10). Thus, unless otherwise illustrated, the truncated number was uniformly chosen as 10 in subsequent studies.

**Table 1:** Convergence of the first four lowest frequency parameters Ω for a perfectly clamped FGV-CNT beam (*V*_*tcnt*_ = 0.12, *L/h* = 10).

*M*	Strong form solution				Rayleigh–Ritz method			
	1st	2nd	3rd	4th	1st	2nd	3rd	4th
4	13.8945	28.2878	44.3428	71.7823	13.8554	27.9426	43.7914	62.2869
5	13.8809	28.0239	44.3052	60.4087	13.8552	27.9151	43.7895	59.6012
6	13.8680	28.0193	43.9247	60.3735	13.8548	27.9151	43.7415	59.5985
7	13.8648	27.9574	43.9195	59.8050	13.8548	27.9118	43.7413	59.4953
8	13.8607	27.9563	43.8183	59.8001	13.8548	27.9118	43.7344	59.4951
9	13.8596	27.9344	43.8169	59.6313	13.8547	27.9111	43.7344	59.4775
10	13.8579	27.9340	43.7781	59.6299	13.8547	27.9111	43.7327	59.4774
11	13.8574	27.9244	43.7776	59.5605	13.8547	27.9108	43.7327	59.4727
12	13.8566	27.9242	43.7595	59.5600	13.8547	27.9108	43.7322	59.4727
13	13.8564	27.9193	43.7593	59.5259	13.8547	27.9108	43.7322	59.4711
14	13.8560	27.9192	43.7497	59.5257	13.8547	27.9108	43.7320	59.4711
15	13.8558	27.9164	43.7496	59.5070	13.8547	27.9107	43.7320	59.4704

With satisfactory results for the convergence studies of the FG-CNTRC beam, which is assumed to feature a perfectly clamped boundary condition at both ends, the numerical validity and rationality of the mentioned definition of the boundary conditions in terms of assigning boundary spring rigidities is evaluated in this section. The first three non-dimensional frequency parameters $\bar{{\rm{\Omega }}}=\omega L\sqrt{{\rho }^{m}[1-{({\upsilon }^{m})}^{2}]/{E}^{m}}$ for FG-CNTRC beams with various CNT distributions and boundary conditions are compared with those reported in the publications of Lin *et al*.^20^ and Yas *et al*.^18^, as shown in Table 2 and Table 3.

**Table 2:** Comparison of the first three frequency parameters $\bar{{\rm{\Omega }}}=\omega L\sqrt{{\rho }^{m}[1-{({\upsilon }^{m})}^{2}]/{E}^{m}}$ for FG-CNTRC beams with various CNT distributions and boundary conditions (*L/h* = 15, *V*_*tcnt*_ = 0.28).

Distributions	Modes	S-S				C-F			
		Lin^20^	Yas^18^	Present		Lin^20^	Yas^18^	Present	
				Strong form	Weak form			Strong form	Weak form
FGV-CNT	1	1.3975	1.4027	1.3639	1.3639	0.4753	0.4761	0.4600	0.4600
	2	3.8370	3.8639	3.7701	3.7703	2.2543	2.2685	2.2106	2.2108
	3	6.6976	6.7618	6.6301	6.6307	4.9590	5.0007	4.8923	4.8933
FGX-CNT	1	1.6409	1.6493	1.6086	1.6086	0.6566	0.6586	0.6405	0.6405
	2	4.4333	4.4752	4.3927	4.3928	2.6763	2.6987	2.6446	2.6448
	3	7.2258	7.3068	7.1907	7.1913	5.5589	5.6150	5.5169	5.5178
UD-CNT	1	1.4348	1.4401	1.3985	1.3981	0.5600	0.5612	0.54320	0.5430
	2	4.1050	4.1362	4.0505	4.0500	2.4449	2.4614	2.4061	2.4056
	3	6.8595	6.9245	6.8086	6.8086	5.2005	5.2446	5.1457	5.1452

**Table 3:** Comparison of the first three dimensionless frequencies $\bar{{\rm{\Omega }}}=\omega L\sqrt{{\rho }^{m}[1-{({\upsilon }^{m})}^{2}]/{E}^{m}}$ for FG-CNTRC beams with various CNT distributions and volume fractions (*L/h* = 15, C-S).

*V*_*tcnt*_	Distributions	C-S								
		Yas			Strong form solution			Rayleigh–Ritz method		
		1st	2nd	3rd	1st	2nd	3rd	1st	2nd	3rd
0.12	UD-CNT	1.2444	3.0159	4.9342	1.2154	2.9668	4.8734	1.2156	2.9671	4.8734
	FGV-CNT	1.1529	2.8472	4.7474	1.1226	2.7932	4.6778	1.1226	2.7931	4.6774
	FGO-CNT	1.0331	2.6814	4.5619	1.0021	2.6224	4.4840	1.0022	2.6227	4.4844
	FGX-CNT	1.3577	3.1817	5.1092	1.3315	3.1383	5.0557	1.3317	3.1386	5.0562
0.17	UD-CNT	1.5602	3.8402	6.3370	1.5214	3.7701	6.2451	1.5217	3.7705	6.2452
	FGV-CNT	1.4344	3.6064	6.0765	1.3949	3.5306	5.9733	1.3949	3.5305	5.9727
	FGO-CNT	1.2769	3.3772	5.8126	1.2374	3.2973	5.7032	1.2375	3.2976	5.7037
	FGX-CNT	1.7188	4.0843	6.6094	1.6834	4.0219	6.5288	1.6836	4.0223	6.5295
0.28	UD-CNT	1.8040	4.3112	6.9987	1.7622	4.2312	6.8867	1.7626	4.2315	6.8867
	FGV-CNT	1.6933	4.1393	6.8633	1.1226	2.7932	4.6778	1.6501	4.0513	6.7355
	FGO-CNT	1.5229	3.9112	6.6127	1.4786	3.8195	6.4808	1.4783	3.8184	6.4775
	FGX-CNT	1.9813	4.6030	7.3560	1.9416	4.5250	7.2448	1.9415	4.5240	7.2418

The geometrical and material constants of the beams are provided as follows: *L/h* = 15, ${E}_{11}^{cnt}=600$ GPa, ${E}_{22}^{cnt}=10$ GPa, ${G}_{12}^{cnt}=17.2$ GPa, *E* ^*m*^ = 2.5 GPa, ${\upsilon }_{12}^{cnt}=0.19$, *v* ^*m*^=0.3, ${\rho }^{cnt}=1400$ kg/m^3^, and ${\rho }^{m}=1190$ kg/m^3^. Table 2 presents the first three non-dimensional frequency parameters $\bar{{\rm{\Omega }}}=\omega L\sqrt{{\rho }^{m}[1-{({\upsilon }^{m})}^{2}]/{E}^{m}}$ for UD-CNT, FGV-CNT and FGX-CNT beams with a total volume fraction *V*_*tcnt*_ = 0.28, and two classical boundary conditions are considered, namely, S-S and C-F. The solutions from the two present numerical approaches are in outstanding agreement with the results from Lin *et al*.^20^ and Yas *et al*.^18^.

The first three dimensionless natural frequencies $\bar{{\rm{\Omega }}}=\omega L\sqrt{{\rho }^{m}[1-{({\upsilon }^{m})}^{2}]/{E}^{m}}$ for FG-CNTRC beams with C-S boundary conditions are presented in Table 3. The results in the present investigation are close to those in the references, and the two present numerical approaches are sufficiently accurate to enable vibrational characterization of FG-CNTRC beams subject to various boundary conditions. Moreover, a further comparison is explored to illustrate the applicability of the linear theories and assumptions in this investigation. Table 4 demonstrates the comparison of natural frequencies between the results calculated by the present method and those reported in studies^54,55^ that adopted first-order beam theory along with von Karman geometric nonlinearity.

**Table 4:** Comparison of the first three frequency parameters Ω of simply supported FG-CNTRC beam with various volume fractions (*L/h* = 25, *h* = 0.01).

*V*_*tcnt*_	Mode number	UD-CNT				FGV-CNT			
		Present		Ansari^54^	Shen^55^	Present		Ansari^54^	Shen^55^
		Strong form	Weak form			Strong form	Weak form		
0.12	1	15.8367	15.8362	15.8569	15.8363	13.4627	13.4553	13.4913	13.5444
	2	51.7703	51.7733	51.8191	51.8139	46.2414	46.1848	46.2767	46.1920
	3	93.5087	93.4972	93.5513	93.8709	86.6740	86.6616	86.7826	86.8513
0.17	1	19.2292	19.2281	19.2565	19.2279	16.2347	16.2333	16.2828	16.2286
	2	64.0863	64.1134	64.1797	64.1381	56.7354	56.7282	56.8608	56.6836
	3	117.5058	117.4909	117.5724	117.8051	108.1623	111.7350	108.3287	108.1428
0.28	1	23.4763	23.4754	23.4954	23.4774	19.9998	19.9986	20.0344	19.9556
	2	74.3537	74.3500	74.3903	74.4687	67.3595	67.3525	67.4387	66.9973
	3	131.4088	131.3941	131.4391	132.2442	124.4277	124.4110	124.5196	123.8009

The first three frequency parameters Ω of simply supported FG-CNTRC beams with different volume fractions are presented in Table 4. Consistency can be observed between the calculated results and the data from the literature. Thus, the mentioned numerical examples indicate that the current solutions possess rapid convergence and satisfactory accuracy, which will be employed for calculating the results for the parametric studies in the following subsections.

### Parametric studies

Tables 2–5 indicate the accuracy and convergence of the present solution. With enhanced confidence in the present solution approach, a variety of further results for FG-CNTRC beams with different boundary conditions and material and geometrical parameters are provided in this section to serve as benchmark solutions for potential studies. In addition, the frequencies calculated by the strong-form solution procedures are close to the results obtained using the Rayleigh–Ritz method. For brevity, only the results obtained from the strong-form solution procedures are included.

**Table 5:** First three frequency parameters Ω for FG-CNTRC beams with various CNT distributions (*L/h* = 10, *V*_*tcnt*_ = 0.12).

Boundary conditions	Modes	CNT distributions				
		UD-CNT	FGΛ-CNT	FGV-CNT	FGX-CNT	FGO-CNT
C-C	1	14.2538	13.8550	13.8550	14.6267	13.4354
	2	28.5113	27.9120	27.9120	29.1546	27.3180
	3	44.3037	43.7355	43.7355	44.9606	43.0775
F-F	1	23.9206	21.9745	21.9745	25.7045	20.3792
	2	40.3698	39.1137	39.1137	41.5025	37.8475
	3	57.7140	56.5360	56.5360	58.8994	55.3918
S-F	1	16.8444	15.4133	15.4133	18.1527	14.2823
	2	34.1859	32.9718	32.9718	35.2902	31.7971
	3	50.5951	49.6210	49.6210	51.5213	48.7128
S-S	1	11.3232	11.1214	11.1214	12.3011	9.4702
	2	27.9037	26.8855	26.8855	28.8111	25.9048
	3	44.0270	43.3760	43.3760	44.7878	42.4459

**Figure 3:**
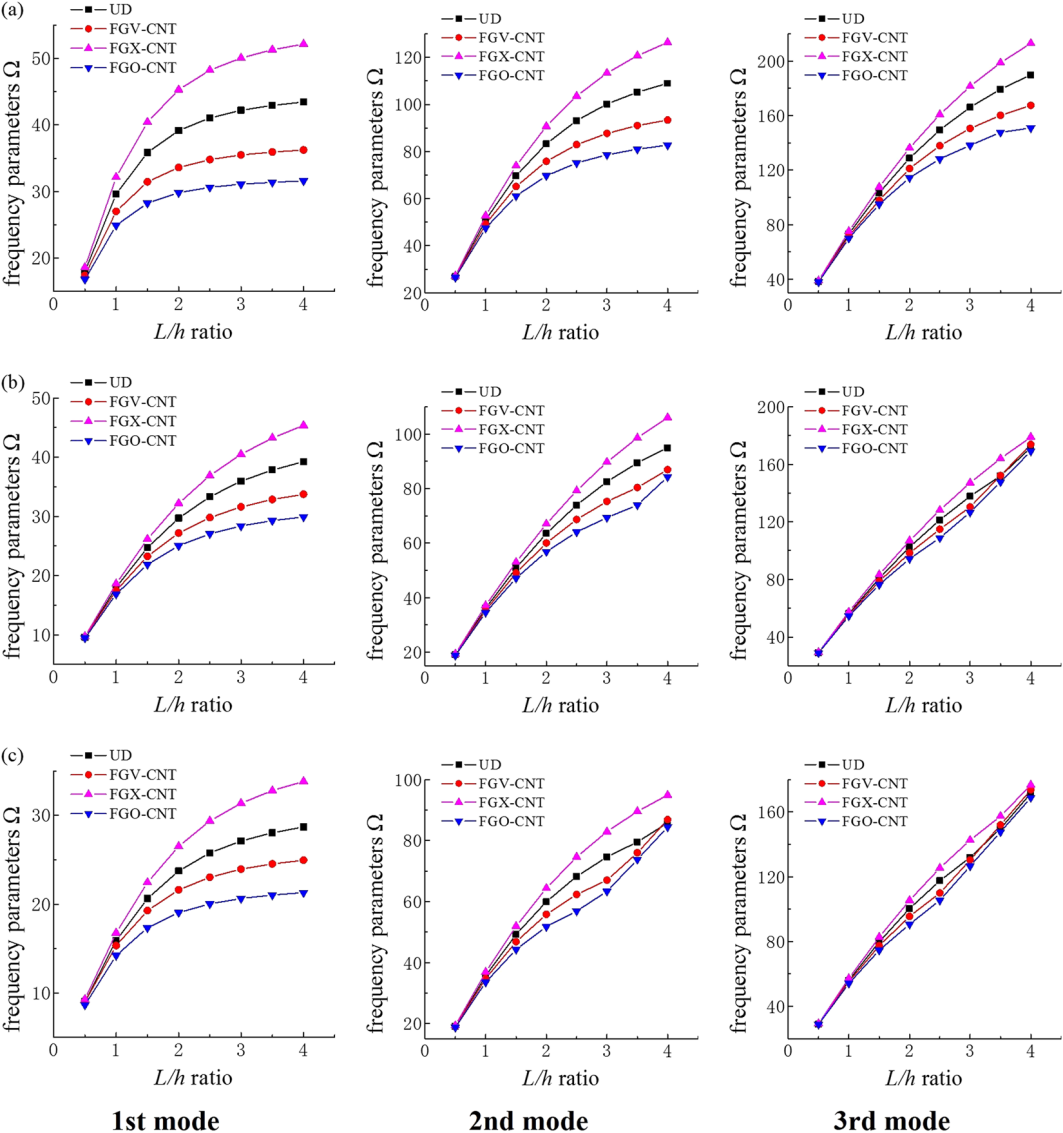
The first three lowest frequency parameters Ω of FG-CNTRC beams with various length-to-thickness ratios. The total CNT volume fraction *V*_*tcnt*_ is equal to 0.17, and the length-to-thickness ratio varies from 0.5 to 4. Three boundary conditions are considered: (**a**) F-F, (**b**) C-C, and (**c**) C-S.

Figure 3 illustrates the relationship between the first three frequency parameters Ω and the length-to-thickness ratio *L/h* for FG-CNTRC beams with various boundary conditions and CNT distributions. The total CNT volume fraction *V*_*tcnt*_ is fixed at 0.17, and the length-to-thickness ratio varies from 0.5 to 4. All dimensionless frequencies increase with increasing length-to-thickness ratio. The fundamental parameter rises gradually with increasing *L/h* ratio; in contrast, the second and third frequency parameters increase sharply. Furthermore, the graphs provide notable results regarding the influence of CNT distributions. The frequency parameters of FG-XCNT beams are always larger than the results of other distributions and beams, with FGO-CNT distributions having the smallest values regardless of the boundary conditions. The same phenomenon can also be found in the following tables.

Table 5 shows the changes in the dimensionless frequencies of FG-CNTRC beams with various CNT distributions. The data in Table 5 lead us to conclude that the CNT distributions have a significant impact on the free-vibration characteristics of FG-CNTRC beams. Furthermore, symmetrical CNT distributions, i.e., FG-XCNT and FG-OCNT, play a notable role in changing the frequency parameters of the CNTRC beams relative to the uniform and asymmetric distributions through the beam thickness.

**Figure 4:**
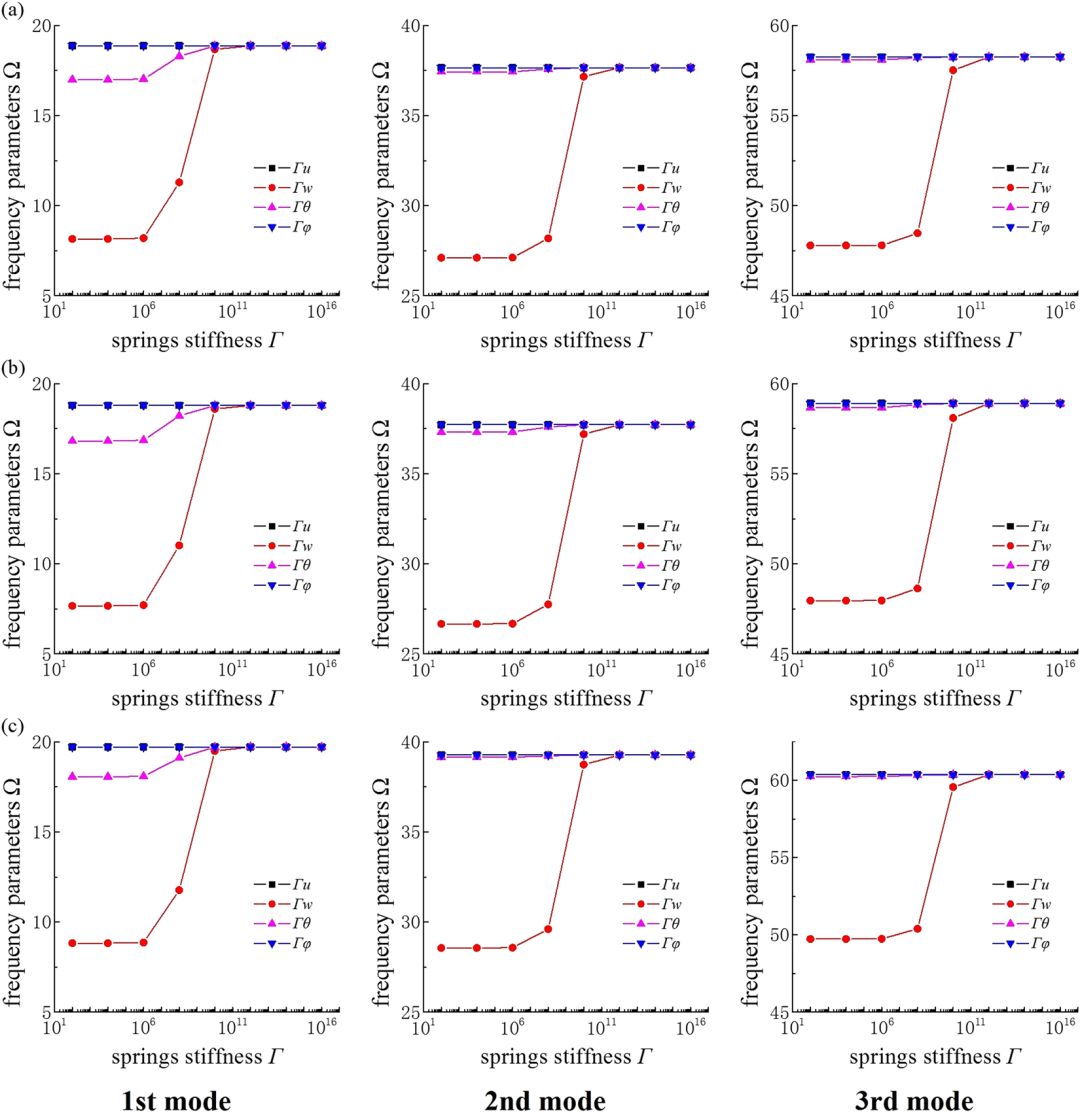
Variation of the first three dimensionless frequencies Ω versus the elastic restraint parameters for FG-CNTRC beams with various CNT distributions: (**a**) UD-CNT, (**b**) FGΛ-CNT, and (**c**) FGX-CNT. The symbols Γ_*u*_, Γ_*w*_, Γ_*θ*_ and Γ_*φ*_ denote the various types of boundary springs. The boundary conditions are considered to be elastically restrained at *x* = 0 and perfectly clamped at *x* = *L*.

One of the primary purposes of this work is to investigate the free-vibration characteristics of FG-CNTRC beams with elastic boundary constraints. Accordingly, Fig. 4 illustrates the effects of four types of boundary spring parameters on the first three frequency parameters of FG-CNTRC beams with elastic supports. The total CNT volume fraction *V*_*tcnt*_ and ratio *L/h* are 0.28 and 10, respectively. The UD-CNT, FGΛ-CNT and FGX-CNT beams are taken into consideration. The symbols Γ_*u*_, Γ_*w*_, Γ_*θ*_ and Γ_*φ*_ are defined to indicate the various kinds of boundary springs, and the boundary condition is defined as elastically restrained only at *x* = 0, where only one group of boundary springs is assigned to variable stiffness values ranging from 10^2^ to 10^16^ and the other groups are assumed to be infinite while the other boundary is clamped at *x* = *L*.

Figure 4 shows that the dimensionless frequency parameters remain stable as the restraint parameters Γ_*u*_ and Γ_*φ*_ change. In contrast, the dimensionless frequencies increase sharply as the restraint parameters Γ_*w*_ and Γ_*θ*_ increase from 10^6^ to 10^10^. Furthermore, there is little variation in the frequency parameters beyond this range. In addition, the results confirm that the influence of the restraint parameter Г_*w*_ is more easily detectable than that of Г_*θ*_. Therefore, the definition of the boundary conditions mentioned above in terms of assigning the values of boundary spring stiffness is reasonable to simulate the real restraints.

The next example is focused on the influence of the total CNT volume fraction on FG-CNTRC beams with classical and elastic boundary conditions. The material properties of the beams are provided as follows: ${E}_{11}^{cnt}=600$ GPa, ${E}_{22}^{cnt}=10$ GPa, ${G}_{12}^{cnt}=17.2$ GPa, *E* ^*m*^ = 2.5 GPa, ${\upsilon }_{12}^{cnt}=0.19$, *v* ^*m*^ = 0.3, ${\rho }^{cnt}=1400$ kg/m^3^, and ${\rho }^{m}=1190$ kg/m^3^. Table 6 indicates the changes in the value of the first three dimensionless frequencies $\bar{{\rm{\Omega }}}=\omega L\sqrt{{\rho }^{m}[1-{({\upsilon }^{m})}^{2}]/{E}^{m}}$ of FG-CNTRC beams as the total CNT volume fraction *V*_*tcnt*_ increases from 0.12 to 0.28. The frequency parameters uniformly increase as the total CNT volume fraction increases.

**Table 6:** First three dimensionless frequencies $\bar{{\rm{\Omega }}}=\omega L\sqrt{{\rho }^{m}[1-{({\upsilon }^{m})}^{2}]/{E}^{m}}$ for FG-CNTRC beams with various total CNT volume fractions (*L/h* = 10).

Distributions	Modes	C-C			F-E2			E1-E1		
		*V*_*tcnt*_			*V*_*tcnt*_			*V*_*tcnt*_		
		0.12	0.17	0.28	0.12	0.17	0.28	0.12	0.17	0.28
UD-CNT	1	1.6491	2.1148	2.3280	2.9960	3.7022	4.1434	2.9914	3.5911	4.0499
	2	3.3231	4.2801	4.6726	4.9598	6.3034	6.8853	4.8392	6.1079	6.7064
	3	5.2064	6.7288	7.2926	6.9132	8.8820	9.6260	6.8542	8.7698	9.5473
FGΛ-CNT	1	1.5838	2.0227	2.2799	2.8056	3.4356	3.9199	2.7912	3.2931	3.7621
	2	3.2315	4.1583	4.6299	4.7946	6.0708	6.7783	4.6535	5.8333	6.5515
	3	5.1083	6.6000	7.2979	6.7713	8.6870	9.6050	6.6681	8.5104	9.4608
FGX-CNT	1	1.7071	2.2055	2.4427	3.1219	3.9621	4.4443	3.1879	3.8920	4.3940
	2	3.4144	4.4220	4.8777	5.0329	6.5356	7.1911	5.0016	6.3703	7.0351
	3	5.3051	6.8933	7.5571	7.0170	9.1109	9.9864	7.0236	9.0436	9.9426
FGO-CNT	1	1.5199	1.9310	2.1956	2.5979	3.2208	3.7011	2.6456	3.0803	3.5206
	2	3.1455	4.0359	4.5151	4.5655	5.8423	6.5693	4.4812	5.5769	6.3101
	3	5.0069	6.4532	7.1648	6.5760	8.4811	9.4203	6.5040	8.2692	9.2386

By introducing the shear correction factor $\kappa$, the first-order shear deformation elasticity theories address the shortcomings of the Euler beam theory, which neglects the effects of transverse shear and rotary inertia. Note that all results in this study are based on the first-order beam theory and that it is necessary to study the influence of the shear correction factor on the free-vibration characteristics of FG-CNTRC beams.

Table 7 presents the fundamental frequency parameters Ω for FG-CNTRC beams in the case in which the shear correction factor increases from 0.1 to 0.9 and compares the results with those calculated by Lin^21^ based on third-order shear deformation elasticity theory. The beam material properties and geometrical parameters are the same as for the FG-CNTRC beams presented in Table 5, where two classical boundary conditions and three types of CNT distribution are considered. The figures reveal that the frequency parameters monotonically increase as the shear correction factor increases from 0.1 to 0.9. To make the frequency parameters consistent with the results based on third-order beam theory, the appropriate shear correction factor $\kappa$ should be selected in calculations with regard to the different boundary conditions.

**Table 7:** Fundamental frequency parameters Ω for FG-CNTRC beams with various shear correction factors.

B.C	Distributions	Shear correction factor									Lin^21^
		0.1	0.2	0.3	0.4	0.5	0.6	0.7	0.8	0.9	
C-C	UD-CNT	5.2652	7.3778	8.9543	10.2477	11.3572	12.3343	13.2099	14.0044	14.7321	12.1067
	FGΛ-CNT	5.2587	7.3378	8.8701	10.1122	11.1656	12.0830	12.8965	13.6271	14.2898	11.9480
	FGX-CNT	5.2976	7.4449	9.0616	10.3993	11.5562	12.5833	13.5109	14.3591	15.1418	12.6733
S-S	UD-CNT	5.0675	6.8610	8.0726	8.9814	9.7000	10.2873	10.7786	11.1970	11.5583	11.3732
	FGΛ-CNT	5.0616	6.8288	8.0108	8.8900	9.5802	10.1408	10.6072	11.0025	11.3424	11.1601
	FGX-CNT	5.1597	7.0759	8.4185	9.4579	10.3027	11.0103	11.6153	12.1407	12.6025	12.3850

**Figure 5:**
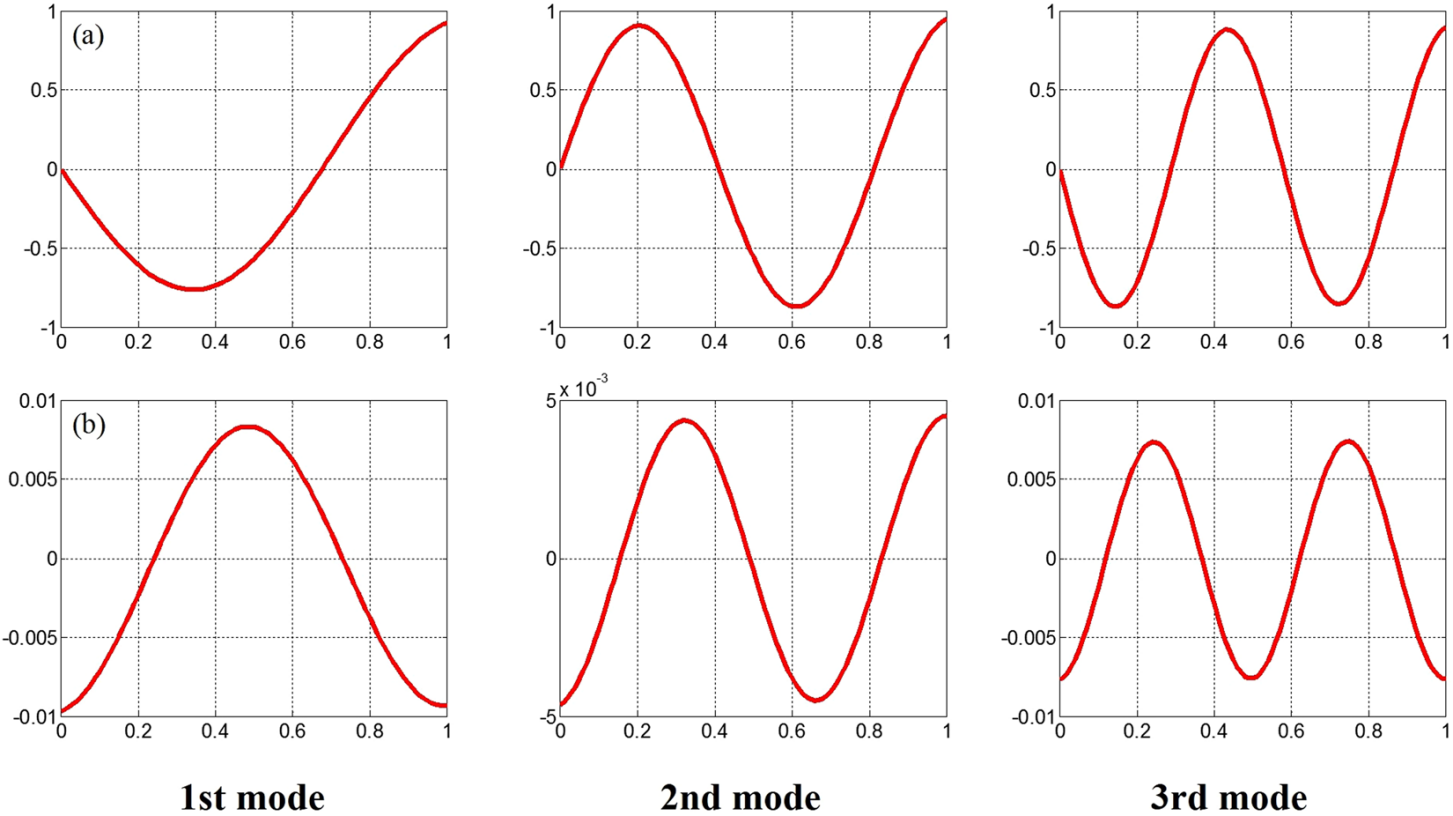
The first three lowest mode shapes of a FGX-CNT beam with two typical elastic boundary conditions. Two beams are taken into account: (**a**) S-E1, *L/h* = 10, *V*_*tcnt*_ = 0.28 and (**b**) E2-E2, *L/h* = 10, *V*_*tcnt*_ = 0.28.

Because the free-vibration results for FG-CNTRC beams with arbitrary boundary conditions are extremely limited in the literature, new results are calculated in Table 8 to provide reference data for practising engineers and to act as a benchmark for potential future studies. Finally, aiming at strengthening our understanding of vibration behaviours of FG-CNTRC beams, several selected mode shapes of the beams addressed in Table 8 are plotted in Fig. 5.

**Table 8:** Fundamental frequency parameters Ω for a FG-CNTRC beam with various boundary conditions.

Distributions	*V*_*tcnt*_	Boundary conditions							
		C-H	C-E1	C-E2	S-E1	F-E1	E1-E1	E1-E2	E2-E2
UD-CNT	0.12	12.6727	20.0666	21.6346	19.7997	26.0059	27.7315	27.7315	28.6638
	0.17	15.8697	23.8453	26.2452	23.2895	31.3209	32.8113	32.8113	34.3048
	0.28	16.9814	25.1491	27.4021	24.9031	33.8933	35.1169	35.1169	36.2263
FGΛ-CNT	0.12	12.2716	19.3900	20.9652	18.8305	24.4182	26.3462	27.5432	27.5432
	0.17	15.3206	22.8423	25.3703	21.8020	29.0142	30.7114	32.6552	32.6552
	0.28	16.8148	24.4339	27.0534	23.7048	32.0973	33.4830	35.1771	35.1771
FGV-CNT	0.12	12.2716	19.3900	20.9652	18.8305	24.4182	26.3462	27.5432	27.5432
	0.17	15.3206	22.8423	25.3703	21.8020	29.0142	30.7114	32.6552	32.6552
	0.28	16.8148	24.4339	27.0534	23.7048	32.0973	33.4830	35.1771	35.1771
FGX-CNT	0.12	13.2801	20.8041	22.2828	20.7267	27.5075	29.0560	29.7225	29.7225
	0.17	16.7565	25.0030	27.2059	24.7818	33.6037	34.9173	35.9925	35.9925
	0.28	18.0569	26.6800	28.6737	26.6242	36.4362	37.5207	38.2933	38.2933
FGO-CNT	0.12	11.4464	18.8696	20.3463	18.1069	23.1569	25.2541	29.7225	26.5735
	0.17	14.2058	22.0359	24.5154	20.7113	27.2019	29.0735	35.9925	31.2253
	0.28	15.7506	23.5927	26.2948	22.5337	30.2494	31.7811	33.8016	33.8016

## Conclusions

An accurate method is developed for the vibration analysis of FG-CNTRC beams. The distribution of CNTs through the thickness of the beam is assumed to vary continuously and smoothly, and five types of distribution, namely, UD-CNT, FGΛ-CNT, FGV-CNT, FGO-CNT and FGX-CNT, are considered. Note that this approach can be uniformly and conveniently applied in vibrational analysis of FG-CNTRC beams with arbitrary boundary conditions, including general elastic boundary conditions. The general boundary conditions can be enforced using the artificial spring technique, in which boundary springs can be assigned any value of stiffness to simulate the real boundary conditions. The energy expressions of the FG-CNTRC beams are written as functions of four displacement components based on first-order shear deformation elasticity theory. Regardless of the boundary conditions, specific geometry and material properties, the displacements and rotational components of the beam are expressed as a superposition of the standard cosine Fourier series and two auxiliary functions. The introduced auxiliary terms are intended to remove potential discontinuous displacement functions and their derivatives at each edge and to ensure the convergence of the series expansions.

By submitting modified Fourier series to governing equations and boundary conditions, the strong-form solution procedure of the modified Fourier method is proposed. For comparison, the Rayleigh–Ritz technique associated with the modified Fourier method is also presented as a weak-form solution. Numerical results obtained by these two present methods are compared with the available results previously reported, and both accuracy and satisfactory convergence are observed. The free-vibration characteristics of FG-CNTRC beams are analysed with a variety of key parameters, for example, the *L/h* ratio, CNT volume fraction, CNT distribution, boundary spring stiffness and shear correction factor. New vibration results containing frequency parameters and mode shapes for the FG-CNTRC beams with classical boundary conditions and elastic supports are calculated to provide reference data for practising engineers and act as a benchmark for future studies.

### Data availability statement

All data generated or analysed during this study are included in this published article.

## Electronic supplementary material

Appendix. Detailed expressions for the total stiffness matrix and mass matrix
