# Supplementary material for: An exact solution for the free-vibration analysis of functionally graded carbon-nanotube-reinforced composite beams with arbitrary boundary conditions
Source: Sci Rep. 2017 Oct 10;7:12909. doi: 10.1038/s41598-017-12596-w (PMC5635134; doi:10.1038/s41598-017-12596-w)
Supplement: Supplementary file 1 — Appendix. Detailed expressions for the total stiffness matrix and mass matrix [file 41598_2017_12596_MOESM1_ESM.pdf]

An exact solution for free vibration analysis of functionally graded carbon nanotube reinforced composite beams with arbitrary boundary conditions

Zeyu Shi[1], Xiongliang Yao[1],Fuzhen Pang[*1], Qingshan Wang[*2,3]

[1] College of Shipbuilding Engineering, Harbin Engineering University, Harbin, 150001, PR China

[2]State Key Laboratory of High Performance Complex Manufacturing, Central South University, Changsha 410083, PR China

[3]College of Mechanical and Electrical Engineering, Central South University, Changsha, 410083, PR China

Appendix. Detailed expressions for the total stiffness matrix and mass matrix

The detailed expressions of the submatrices $\mathbf{K}_{ij}$ and $\mathbf{M}_{ij}$ in Eq. (33) are provided as follows.

$$\mathbf{K}_{uu} = \int_0^L \left(\bar{A}_{11} \frac{\partial \mathbf{H}^T}{\partial x} \frac{\partial \mathbf{H}}{\partial x} \right) dx + k_0^u \mathbf{H}^T \mathbf{H}\Big|_{x=0} + k_L^u \mathbf{H}^T \mathbf{H}\Big|_{x=L}$$

$$\mathbf{K}_{u\theta} = \int_0^L \left(\bar{B}_{11} \frac{\partial \mathbf{H}^T}{\partial x} \frac{\partial \mathbf{H}}{\partial x} \right) dx$$

$$\mathbf{K}_{u\phi} = \int_0^L \left(\bar{B}_{16} \frac{\partial \mathbf{H}^T}{\partial x} \frac{\partial \mathbf{H}}{\partial x} \right) dx$$

$$\mathbf{K}_{ww} = \int_0^L \left(A_{55} \frac{\partial \mathbf{H}^T}{\partial x} \frac{\partial \mathbf{H}}{\partial x} \right) dx + k_0^w \mathbf{H}^T \mathbf{H}\Big|_{x=0} + k_L^w \mathbf{H}^T \mathbf{H}\Big|_{x=L}$$

$$\mathbf{K}_{w\theta} = \int_0^L \left(A_{55} \frac{\partial \mathbf{H}^T}{\partial x} \mathbf{H} \right) dx$$

$$\mathbf{K}_{\theta\theta} = \int_0^L \left(\bar{D}_{11} \frac{\partial \mathbf{H}^T}{\partial x} \frac{\partial \mathbf{H}}{\partial x} + A_{55} \mathbf{H}^T \mathbf{H} \right) dx + k_0^\theta \mathbf{H}^T \mathbf{H}\Big|_{x=0} + k_L^\theta \mathbf{H}^T \mathbf{H}\Big|_{x=L}$$

$$\mathbf{K}_{\theta\phi} = \int_0^L \left(\bar{D}_{16} \frac{\partial \mathbf{H}^T}{\partial x} \frac{\partial \mathbf{H}}{\partial x} \right) dx$$

$$\mathbf{K}_{\phi\phi} = \int_0^L \left(\bar{D}_{66} \frac{\partial \mathbf{H}^T}{\partial x} \frac{\partial \mathbf{H}}{\partial x} \right) dx + k_0^\phi \mathbf{H}^T \mathbf{H}\Big|_{x=0} + k_L^\phi \mathbf{H}^T \mathbf{H}\Big|_{x=L}$$

$$\mathbf{M}_{uu} = \mathbf{M}_{ww} = \int_0^L \left(I_1 \mathbf{H}^T \mathbf{H} \right) dx$$

$$\mathbf{M}_{u\theta} = \int_0^L \left(I_2 \mathbf{H}^T \mathbf{H} \right) dx$$

$$\mathbf{M}_{\theta\theta} = \mathbf{M}_{\phi\phi} = \int_0^L \left(I_3 \mathbf{H}^T \mathbf{H} \right) dx$$

$$\mathbf{H} = \begin{bmatrix} \mathbf{H}_f & \mathbf{H}_a \end{bmatrix}$$
